# Metabolic profiling of *Chimonanthus grammatus via* UHPLC-HRMS-MS with computer-assisted structure elucidation and its antimicrobial activity

**DOI:** 10.3389/fpls.2023.1138913

**Published:** 2023-05-09

**Authors:** Haibo Hu, Volkan Tekin, Bin Hu, Mahdi Yaghoobi, Ajmal Khan, Alokesh Kumar Ghosh, Sujogya Kumar Panda, Hao Huang, Walter Luyten

**Affiliations:** ^1^ National Engineering Research Center for Modernization of Traditional Chinese Medicine - Hakka Medical Resources Branch, School of Pharmacy, Gannan Medical University, Ganzhou, China; ^2^ Animal Physiology and Neurobiology Section, Department of Biology, KU Leuven, Leuven, Belgium; ^3^ Department of Phytochemistry, Medicinal Plants and Drug Research Institute, Shahid Beheshti University, Tehran, Iran; ^4^ Leishmania Diagnostic & Drug Delivery Research Laboratory, University of Peshawar, Peshawar, Pakistan; ^5^ Fisheries and Marine Resource Technology Discipline, Khulna University, Khulna, Bangladesh; ^6^ Center of Environment Climate Change and Public Health, Utkal University, Vani Vihar, Bhubaneswar, Odisha, India

**Keywords:** *Chimonanthus*, *Chimonanthus grammatus*, MS Fragmenter, ChromGenius, isofraxidin, kaempferol, quercitrin

## Abstract

*Chimonanthus grammatus* is used as Hakka traditional herb to treat cold, flu, etc. So far, the phytochemistry and antimicrobial compounds have not been well investigated. In this study, the orbitrap-ion trap MS was used to characterize its metabolites, combined with a computer-assisted structure elucidation method, and the antimicrobial activities were assessed by a broth dilution method against 21 human pathogens, as well as the bioassay-guided purification work to clarify its main antimicrobial compounds. A total of 83 compounds were identified with their fragmentation patterns, including terpenoids, coumarins, flavonoids, organic acids, alkaloids, and others. The plant extracts can strongly inhibit the growth of three Gram-positive and four Gram-negative bacteria, and nine active compounds were bioassay-guided isolated, including homalomenol C, jasmonic acid, isofraxidin, quercitrin, stigmasta-7,22-diene-3*β*,5*α*,6*α*-triol, quercetin, 4-hydroxy-1,10-secocadin-5-ene-1,10-dione, kaempferol, and *E*-4-(4,8-dimethylnona-3,7-dienyl)furan-2(5H)-one. Among them, isofraxidin, kaempferol, and quercitrin showed significant activity against planktonic *Staphylococcus aureus* (IC_50 _= 13.51, 18.08 and 15.86 µg/ml). Moreover, their antibiofilm activities of *S. aureus* (BIC_50 _= 15.43, 17.31, 18.86 µg/ml; BEC_50 _= 45.86, ≥62.50, and 57.62 µg/ml) are higher than ciprofloxacin. The results demonstrated that the isolated antimicrobial compounds played the key role of this herb in combating microbes and provided benefits for its development and quality control, and the computer-assisted structure elucidation method was a powerful tool for chemical analysis, especially for distinguishing isomers with similar structures, which can be used for other complex samples.

## Graphical Abstract

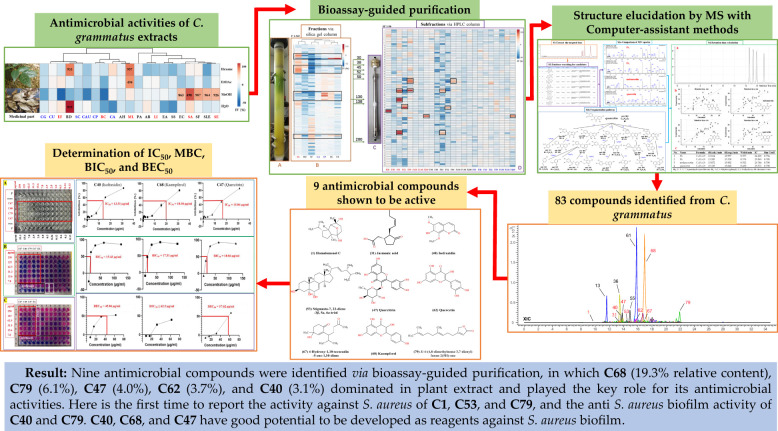

## 1 Introduction

As a Hakka herb, *Chimonanthus grammatus* M.C. Liu (ChG) is from the Calycanthaceae family and only distributed in Ganzhou, Jiangxi Province, China. The local Hakka people have cultivated it for decades and utilized it as a traditional herb with effects on cold, flu, cough, and rheumatic arthritis (Jiang et al., 2015; Hu et al., 2022). There is a tradition to put the tender stem and leaves inside fishpond for protecting fish against infections (Liu et al., 2011), indicating the plant should have antimicrobial activities and related active compounds. Although ChG was documented as a new species in 1984, most local people treat it as other local *Chimonanthus* plants, such as *C. praecox*, *C. salicifolius*, or *C. nitens*, which have much longer history as herbal medicine (Flora of China Editorial Committee, 2008). *Chimonanthus* plants have been developed as several medicaments, such as ‘Shan la mei ke li’ (granules for wind-heat type common cold, fever, chills, sore throat, etc.), ‘La mei you’ (oil for skin ulcer, eczema, swelling pain, etc.).

*Chimonanthus* is only distributed in south China, and by now most research works have been done for these common species, from which more than one hundred compounds were isolated, including alkaloids, sesquiterpenoids, coumarins, flavonoids, organic acids, etc. (Xiao and Liu, 2003; Deng et al., 2004; Wang et al., 2011; Li and Zou, 2018; Shuaifeng and Zhengrong, 2018; Shu et al., 2019a; Guo et al., 2021), exhibiting antimicrobial, anti-tumor, anti-inflammatory, antioxidant, anti-lipids, hypoglycemia, anti-depression, liver protection, cough suppressant, anti-diarrhea, and other biological activities (Lingyun et al., 2012; Bingkun and Yaoming, 2003; Zhang et al., 2016; Zhang et al., 2017; Ye et al., 2020). *Chimonanthus* plants, like *C. praecox*, also showed some toxicity against rats, rabbits, cattle, or goats, with possible kidney damage, causing hematuria, which was reported as the reason for its alkaloids. However, rare studies have been done on ChG, such as genetic diversity and comparison studies (Jiang et al., 2012; Jiang et al., 2013; Jiang et al., 2015), the essential oil and its activity (Liu et al., 2011), and several isolated non-volatile compounds (Wang, 2012; Ling, 2014; Ling and Zou, 2014; Zhang et al., 2017; Li et al., 2020). By now, the chemical profiling of ChG and its antimicrobial compound basis have still not been clearly elucidated. Therefore, we focused on a comprehensive study investigating the chemical profile *via* UHPLC-HRMS-MS, antimicrobial activities against various human pathogens, and identification of the main antimicrobial compounds *via* bioassay-guided purification. In addition, for the active extracts and abundant compounds, we determined their IC_50_, MBC, and antibiofilm activities to help assess their medicinal potentials.

## 2 Materials and methods

### 2.1 Botanical sample preparation and reagents

Herbal materials were collected from Geyou forestry station (25°28’ N, 115°44’ E) and Maogongfa hill (25°29’ N, 115°45’ E), Anyuan County, Ganzhou, China. The plant was identified as *Chimonanthus grammatus* M.C. Liu according to the Flora of China and http://www.theplantlist.org. The voucher specimens (No.GZ1805) were stored in the Herbarium of Chinese Medicine at Gannan Medical University. The reagents included water, DMSO, formic acid, trifluoroacetic acid, acetonitrile, methanol, ethyl acetate, hexane, antibacterial controls (ciprofloxacin and chloramphenicol), antifungal control (miconazole), and antibiofilm control (sodium dodecyl sulfate). The stem and leaves, as well as the reagents, were prepared with the same methods and resources as previously described (Hu et al., 2021a). Resazurin salt was from Acros Organics (Lot# AC189900010, Geel, Belgium). A reference compound, quercetin was purchased from Sigma-Aldrich (Lot#SLBM7336V, Darmstadt, Germany).

### 2.2 Antimicrobial and antibiofilm testing

The crude extracts and separated fractions were assessed for their antimicrobial activities by a broth microdilution method in 96-well microtiter plates, and the IV (inhibition value %), IC_50_ (concentration inhibiting growth by 50%) calculations, and heat map analysis were performed as described before (Hu et al., 2021a). The 21 human pathogens for assessing the extracts included six Gram-positive (G^+^) bacteria [SE: *Staphylococcus epidermidis* (ATCC 1457), SA: *Staphylococcus aureus* (ATCC6538, Rosenbach), LI: *Listeria innocua* (LMG 11387), EF: *Enterococcus faecalis* (HC-1909-5), ML: *Micrococcus luteus* (DPMB 3) and BC: *Bacillus cereus* (LMG9610)], nine Gram-negative (G^–^) bacteria [PA: *Pseudomonas aeruginosa* (PAO1), EC: *Escherichia coli* (ATCC 47076), SS: *Shigella sonnei* (LMG 10473), SF: *Shigella flexneri* (LMG10472), EA: *Enterobacter aerogenes* (ATCC 13048), AB: *Acinetobacter baumannii* (RUH134), SLE: *Salmonella enterica* subsp. *enterica* (ATCC 13076), BD: *Brevundimonas diminuta* (from Prof. Rob Lavigne at KU Leuven) and AH: *Aeromonas hydrophila* (ATCC 7966)], and six fungi [CP: *Candida parapsilosis* (ATCC 22019), CA: *C. albicans* (SC 5314), CG: *C. glabrata* (ATCC 2001), CAU: *C. auris* (OS299), CU: *C. utilis* (IHEM 4005), and SC: *Saccharomyces cerevisiae* (ATCC 7754)]. The abundant pure compounds were also tested against a biofilm strain: *Staphylococcus aureus* (USA 300). The preformed biofilm and biofilm formation activities were tested for BIC_50_ (concentration inhibiting biofilm formation by 50%), and BEC_50_ (concentration required to eradicate 50% of a preformed biofilm) as described before (Kipanga et al., 2020).

### 2.3 Isolation and purification

The large-scale extracts were prepared by a sonication assistance method in methanol and ethyl acetate separately, then combined as the final ChG extract. The mixture of extraction was separated *via* a silica gel column in a similar way as the previous study (Hu et al., 2021a). In brief, 6.4 g of dried extracts (obtained from 200 g plant materials) was used for the elution by the mobile phases consisting of hexane (A), ethyl acetate (B), methanol (C), and 25% acetic acid in methanol (D) with a gradient from 95% A and 5% B to 100% D (5%-10% difference per 10 min, flow rate: 40 mL/min). Then 242 fractions were collected. The eight most active fractions were selected for HPLC separation and compound purification with semi-preparative C_18_ column using conditions summarized in **Table 1**.

**Table 1:** HPLC gradients of selected fractions.

Fractions	Dissolved in ACN%	Gradients
F30	85%	0-5 min, 20% B; 8-55 min, 20% to 60% B; 55-65 min, 60% to 100% B; 65-80 min, 100% B.
F38	85%	0-5 min, 10% B; 5-10 min, 10% to 40% B; 10-65 min, 40% to 100% B; 65-80 min, 100% B.
F45	80%	0-8 min, 10% B; 8-20 min, 10% to 30% B; 20-80 min, 30% to 35% B.
F52	75%	0-8 min, 10% B; 8-62 min, 10% to 65% B; 62-72 min, 65% to 100% B; 72-80 min, 100% B.
F59	75%	0-8 min, 10% B; 8-62 min, 10% to 60% B; 62-72 min, 60% to 100% B; 72-80 min, 100% B.
F130	15%	0-8 min, 10% B; 8-60 min, 10% to 60% B; 60-70 min, 60% to 100% B; 70-80 min, 100% B.
F138	15%	0-8 min, 5% B; 8-65 min, 5% to 40% B; 65-72 min, 40% to 100% B; 72-80 min, 100% B.
F200	10%	0-8 min, 5% B; 8-65 min, 5% to 30% B; 65-72 min, 30% to 100% B; 72-80 min, 100% B.

### 2.4 UHPLC-HRMS-MS condition and data analysis

The MS samples were randomly sampled from the extract and redissolved in MeOH at 0.1 mg/mL, then 5 μl of 20-times dilution was injected for each MS detection. An Ultimate 3000 UHPLC system coupled to an ultra-high resolution Orbitrap Elite hybrid mass spectrometer was used. The equipment setting, data analysis, and heat map were done as described before (Hu et al., 2021a), from which small changes were made for the MS optimization like the previous study (Hu et al., 2021b), as follows: full scan mode with 100-1500 *m/z* mass range, MS/MS fragmentation for top 10 precursors (dd-MS^2^-TOP 10) with repetition in the range of 100-1500 *m/z*. The MS data were analyzed by the software packages, Xcalibur 4.2, Freestyle™ 1.5, ACD/MS Workbook Suite 2021 with MS Fragmenter, and ChromGenius.

## 3 Results and discussion

### 3.1 Chemical profiling of ChG

**Figure 1:**
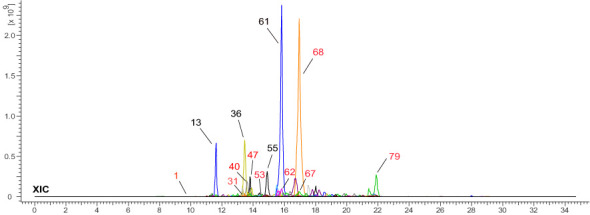
Total ion chromatogram of *Chimonanthus grammatus*, and the main components (marked with the same number as in **Table 2**).

The MS data of ChG extract was automatically matched with a manually built database *via* ACD/MS Workbook Suite. The total ion chromatogram (TIC) of the plant extract was extracted by the matched ions and the results of extracted ion chromatogram (EIC) in positive ion mode were shown as an example in **Figure 1**. The identified compounds and their fragmentations were listed in **Table 2**. A total of 83 components were tentatively identified, including 21 terpenoids, 20 coumarins, 19 flavonoids, 15 organic acids, 4 alkaloids, 2 glycosides, 1 anthraquinone, and 1 steroid. Of note, 5,6,7-trimethoxycoumarin and kaempferol had the highest relative contents (23% and 19%, respectively) according to the peak area comparison, and the other main components with more than 2% contents are *E*-4-(4,8-dimethylnona-3,7-dienyl)furan-2(5H)-one, scopoletin, nicotiflorin, scoparone, quercitrin, quercetin, isofraxidin, fraxetin, luteolin, cucumin E, and melanoxetin. To date, this is the first report on the chemical profiling of ChG non-volatile compounds by mass spectrometry, as well as the fragmentation patterns of most compounds here.

**Table 2:** MS identification of compounds from *Chimonanthus grammatus*.

No.	Name	RT	Formula	Ion Mode	Calculated *m*/*z*	Observed *m*/*z*	Diff. (ppm)	MS/MS Fragments	RC ^a^ (%)
1	**Homalomenol C**	9.90	C_15_H_26_O_3_	[M+H]^+^	255.1955	255.1955	0.00	237.2567, 219.2709, 201.3904, 163.2317, 159.2447, 95.2578	0.043
				[M-H]-	253.1810	253.1806	-1.58	235.0760, 225.8991, 209.1213, 191.1002, 183.1498, 164.9577	
2	Casimiroedine	10.29	C_21_H_27_N_3_O_6_	[M+H]^+^	418.1973	418.1973	0.00	401.3553, 282.1857, 265.1134, 193.9913, 137.0768	0.090
3	Methyl *β*-hydroxy-benzoate	10.36	C_8_H_8_O_3_	[M+H]^+^	153.0546	153.0546	0.00	135.1994, 125.0194, 109.0685, 93.1375	0.003
4	Chlorogenic acid	10.80	C_16_H_18_O_9_	[M+H]^+^	355.1024	355.1022	-0.56	337.4137, 285.1088, 266.9599, 193.0769, 163.1834	0.002
5	Methyl 4-*β*-d-glucopyranosyl-ferulate	10.94	C_17_H_22_O_9_	[M+H]^+^	371.1337	371.1331	-1.62	355.1042, 311.5054, 209.0489, 179.1788, 147.2108	0.001
6	Fraxin/7-Hydroxy-6-methoxy-2-oxo-2H-chromen-8-yl *α*-*L*-galactopyranoside	11.03	C_16_H_18_O_10_	[M+H]^+^	371.0973	371.0974	0.27	355.1338, 226.9893, 217.4950, 209.0205, 163.2549	0.229
7	(+)-Calycanthine/(-)-calycanthine/(-)iso-calycanthine	11.22	C_22_H_26_N_4_	[M+H]^+^	347.2230	347.2231	0.29	304.3145, 290.1706, 285.3776, 273.2586, 173.2570	0.748
8	Meratin	11.29	C_27_H_30_O_17_	[M+H]^+^	627.1556	627.1559	0.48	609.1452, 595.1294, 567.1346, 447.0924, 325.1132, 163.0604	0.014
9	Scopolin	11.31	C_16_H_18_O_9_	[M+H]^+^	355.1024	355.1021	-0.84	267.2575, 251.4835, 193.0445, 163.0500	0.259
10	Isoscopoletin	11.33	C_10_H_8_O_4_	[M+H]^+^	193.0495	193.0493	-1.04	178.2558, 165.1324, 161.0099, 149.3918, 133.0560, 117.0862	0.565
11	Methylparaben	11.61	C_8_H_8_O_3_	[M+H]^+^	153.0546	153.0547	0.65	135.2316, 125.0681, 121.0431, 109.1384, 93.0598	0.015
12	(+)-Vomifoliol	11.61	C_13_H_20_O_3_	[M+H]^+^	225.1485	225.1488	1.33	207.1083, 183.2634, 167.0187, 149.2507, 123.1946, 109.2451	0.060
13	Fraxetin	11.63	C_10_H_8_O_5_	[M+H]^+^	209.0444	209.0445	0.48	194.2644, 181.1243, 177.2570, 163.1661, 149.1060	2.929
14	Kuromanin	11.67	C_21_H_21_O_11_^+^	[M]^+^	449.1078	449.1079	0.22	431.4693, 413.4693, 353.2398, 287.1261	0.017
15	Calycanthoside	11.69	C_17_H_20_O_10_	[M+H]^+^	385.1129	385.1130	0.26	367.1024, 353.0867, 335.0761, 325.0918, 163.0601	0.003
16	Benzyl alcohol *α*-*L*-rhamnopyranosyl (1→6)-*β*-D-glucopyranoside/Phenethyl alcohol-*β*-D-xylopyranosyl (1→6)-*β*-D-glucopyranoside	11.78	C_19_H_28_O_10_	[M+H]^+^	417.1755	417.1751	-0.96	399.3967, 385.2809, 329.2489, 255.1477, 194.1008,	0.002
17	Tomenin	11.97	C_17_H_20_O_10_	[M+H]^+^	385.1129	385.1131	0.52	367.1026, 349.0918, 325.0920, 179.0550, 163.0602	0.001
18	(3-Acetyl-6-acetyloxy-7-hydroxy-7-methyl-4-propan-2-yl-1,2,3,3*a*,4,5,6,7*a*-octahydroinden-5-yl) acetate	11.99	C_19_H_30_O_6_	[M+H]^+^	355.2115	355.2120	1.41	337.4359, 301.1819, 267.0030, 211.2492, 193.2132, 163.1808	0.038
19	Syringic acid	12.08	C_9_H_10_O_5_	[M+H]^+^	199.0601	199.0599	-1.00	199.0601, 181.0498, 166.0266, 151.0392, 122.0367	0.010
20	Cyanidin 3-*O*-rutinoside	12.12	[C_27_H_31_O_15_]^+^	[M]^+^	595.1657	595.1661	0.67	577.5649, 449.0775, 433.4061, 432.6970, 415.2662, 287.1130	0.034
21	*p*-Coumaroylquinic acid	12.33	C_16_H_18_O_8_	[M+H]^+^	339.1074	339.1076	0.59	321.3571, 311.1271, 273.2238, 176.9738, 146.9915	0.147
22	3-Hydroxy-5,6-epoxy-*β*-ionol 3-*O*-*β*-D-glucopyranoside	12.35	C_19_H_32_O_8_	[M+H]^+^	389.2170	389.2172	0.51	371.2066, 328.1887, 316.1520, 163.0609	0.013
23	Pisonin C	12.42	C_10_H_8_O_5_	[M+H]^+^	209.0444	209.0445	0.48	194.2037, 181.2864, 163.2250, 148.9003, 107.2939	0.684
24	trans-*p*-Coumaraldehyde	12.63	C_9_H_8_O_2_	[M+H]^+^	149.0597	149.0599	1.34	131.2086, 121.1665, 107.1090, 93.6356, 79.1051	0.013
25	7-Hydroxy-4-isopropyl-6-methylcoumarin	12.72	C_13_H_14_O_3_	[M+H]^+^	219.1016	219.1017	0.46	201.2499, 189.1153, 174.2192, 159.3086, 131.2345	0.289
26	Salifoxacine A/Salifoxacine B	12.93	C_22_H_26_N_4_O	[M+H]^+^	363.2179	363.2186	1.93	345.2668, 332.4304, 175.2455, 173.1292, 130.1643	0.360
27	2’,4’,5,7,8-Pentahydroxyisoflavone	13.00	C_15_H_10_O_7_	[M+H]^+^	303.0499	303.0500	0.33	285.0612, 257.1276, 229.1588, 201.2081, 165.1519	0.952
28	Rutin	13.02	C_27_H_30_O_16_	[M+H]^+^	611.1607	611.1608	0.16	575.5147, 465.2427, 449.2732, 303.0549	1.468
29	*p*-Coumaric acid	13.08	C_9_H_8_O_3_	[M+H]^+^	165.0546	165.0547	0.61	165. 0556, 147.0444, 130.0416, 118.0420, 102.0464	0.028
30	Caffeic acid	13.21	C_9_H_8_O_4_	[M+H]^+^	181.0495	181.0498	1.66	163.1955, 153.2260, 139.1021, 135.1369, 121.0201	0.052
31	**Jasmonic acid**	13.29	C_12_H_18_O_3_	[M+H]^+^	211.1329	211.1330	0.47	193.1317, 183.0209, 165.3889, 123.1810, 95.2296	0.183
				[M-H]-	209.1180	209.1187	3.35	165.1991, 163.0641, 124.9956, 95.0152, 82.8971	
32	Isoquercitrin	13.31	C_21_H_20_O_12_	[M+H]^+^	465.1028	465.1029	0.22	447.3377, 429.3558, 415.5456, 399.2437, 367.5966, 343.0088, 303.0803	0.822
33	3-(3,4-Dihydroxyphenyl)-2,6,8-trihydroxy-4H-chromen-4-one	13.33	C_15_H_10_O_7_	[M+H]^+^	303.0499	303.0500	0.33	303.2198, 257.1329, 229.1967, 165.1119, 153.0812	1.352
34	Scopoletin	13.45	C_10_H_8_O_4_	[M+H]^+^	193.0495	193.0497	1.04	178.1470, 165.1160, 160.9847, 147.2249, 133.1324	5.484
35	Luteoloside	13.45	C_21_H_20_O_11_	[M+H]^+^	449.1078	449.1086	1.78	431.4451, 395.3226, 384.2781, 329.3301, 287.1150	0.354
36	Nicotiflorin	13.45	C_27_H_30_O_15_	[M+H]^+^	595.1657	595.1665	1.34	449.3987,433.5779, 431.4893, 287.1202, 213.2154	5.072
37	2,6,2’,6’-Tetra-methoxy-4,4’-bis (2,3-epoxy-1-hydroxyl-propyl)-biphenyl	13.49	C_22_H_26_O_8_	[M+H]^+^	419.1700	419.1695	-1.19	401.2791, 282.2223, 208.0349, 194.0492, 136.9383	0.015
38	Narcissin	13.51	C_28_H_32_O_16_	[M+H]^+^	625.1763	625.1758	-0.80	607.1663, 565.1555, 461.1083, 309.1187, 147.0654	0.004
39	7-Hydroxy-6-methoxych roman-2-one	13.53	C_10_H_10_O_4_	[M+H]^+^	195.0652	195.0650	-1.03	177.0824, 135.1149, 133.0601, 121.0901, 81.0978	0.026
40	**Isofraxidin**	13.59	C_11_H_10_O_5_	[M+H]^+^	223.0601	223.0599	-0.90	208.0067, 195.2093, 191.4253, 181.4583, 162.9882	3.083
				[M-H]-	221.0460	221.0461	0.45	205.0729, 176.8949, 149.0305, 126.8071	
41	Cleomiscosin B	13.66	C_20_H_18_O_8_	[M+H]^+^	387.1074	387.1073	-0.26	369.2598, 298.2961, 234.8281, 225.2734, 207.3047	0.061
42	Astralgin	13.66	C_21_H_20_O_11_	[M+H]^+^	449.1078	449.1077	-0.22	383.4879, 353.0158, 287.0600	0.999
43	Cleomiscosin C	13.68	C_21_H_20_O_9_	[M+H]^+^	417.1180	417.1180	0.00	399.5276, 389.4977, 282.3288, 255.1120, 194.0420, 165.4308	0.055
44	(-)-Loliolide	13.70	C_11_H_16_O_3_	[M+H]^+^	197.1172	197.1173	0.51	179.2507, 160.6769, 135.2392, 107.1514	0.324
45	Luteolin	13.80	C_15_H_10_O_6_	[M+H]^+^	287.0550	287.0552	0.70	287.0794, 258.2573, 213.2696, 153.1762, 141.2531	2.419
46	meso-Chimonanthine/Chimonanthine	13.80	C_22_H_26_N_4_	[M+H]^+^	347.2230	347.2226	-1.15	316.2624, 290.3053, 285.1615, 237.2980, 173.0902, 144.1213	0.049
47	**Quercitrin**	13.80	C_21_H_20_O_11_	[M+H]^+^	449.1078	449.1081	0.67	431.3559, 303.2665, 287.1027	4.000
				[M-H]-	447.0930	447.0931	0.22	429.0628, 367.3325, 327.0300, 300.9843, 284.0090, 254.9638, 198.8138	
48	Blumenol C	13.88	C_13_H_22_O_2_	[M+H]^+^	211.1693	211.1693	0.00	193.1203, 169.2938, 133.2207, 105.1861, 91.0543	0.049
49	Melanoxetin	13.90	C_15_H_10_O_7_	[M+H]^+^	303.0499	303.0498	-0.33	303.2515, 257.1025, 229.2363, 164.9839	2.188
50	Luteolin 7-*O*-(6-*O*-Malonyl-*β*-D-Glucoside)	14.36	C_24_H_22_O_14_	[M+H]^+^	535.1082	535.1088	1.12	517.2321, 395.1935, 329.2661, 287.1368	0.422
51	Chimsalicifoliusin B	14.48	C_20_H_14_O_8_	[M+H]^+^	383.0761	383.0764	0.78	351.2394, 319.2092, 267.1504, 201.0965, 160.0562	0.008
53	**Stigmasta-7,22-diene-3*β*,5*α*,6*α*-triol**	14.50	C_28_H_46_O_3_	[M+H]^+^	431.3520	431.3521	0.23	413.4407, 385.3807, 343.2612, 311.4564, 277.4949	0.013
54	Emodin-8-*O*-*β*-D-glucopyranoside	14.52	C_21_H_20_O_10_	[M+H]^+^	433.1129	433.1136	1.62	397.2943, 329.1075, 287.2781, 271.3486, 144.1353	0.520
54	Yingzhaosu C	14.82	C_15_H_22_O_3_	[M+H]^+^	251.1642	251.1643	0.40	233.2981, 215.0963, 205.5200, 161.0398	1.693
55	Scoparone	14.89	C_11_H_10_O_4_	[M+H]^+^	207.0652	207.0653	0.48	191.4636, 179.1335, 175.1765, 164.1226, 151.1009	4.232
56	Benzyl acetate	14.90	C_9_H_10_O_2_	[M+H]^+^	151.0754	151.0755	0.66	133.1454, 123.0562, 109.0509, 107.0399, 81.1841	0.049
57	trans-Cinnamic acid	15.40	C_9_H_8_O_2_	[M+H]^+^	149.0597	149.0598	0.67	121.2588, 107.1200, 103.1292, 93.0771	0.036
58	6,7,8-Trimethoxycoumarin	15.45	C_12_H_12_O_5_	[M+H]^+^	237.0757	237.0758	0.42	222.0765, 209.1855, 193.0954, 176.1606, 149.2395	0.402
59	Ethylparaben	15.53	C_9_H_10_O_3_	[M+H]^+^	167.0703	167.0701	-1.20	149.0444, 125.0413, 121.1554, 107.1059, 95.7185	0.082
60	Cleomiscosin A	15.78	C_20_H_18_O_8_	[M+H]^+^	387.1074	387.1076	0.52	369.2173, 357.1235, 337.2031, 263.2182, 161.2504	0.186
61	5,6,7-Trimethoxycoumarin	15.82	C_12_H_12_O_5_	[M+H]^+^	237.0757	237.0757	0.00	222.0461, 209.0504, 204.0299, 193.1172, 176.0104	23.311
62	**Quercetin**	15.84	C_15_H_10_O_7_	[M+H]^+^	303.0499	303.0498	-0.33	303.3013, 285.2981, 257.3037, 229.0610, 164.9685, 137.1835	3.694
				[M-H]-	301.0350	301.0356	1.99	282.9657, 272.9723, 256.9994, 206.9231, 162.9725	
63	4,4’-Biisofraxidin/3,3’-Biisofraxidin	16.40	C_22_H_18_O_10_	[M+H]^+^	443.0973	443.0972	-0.23	428.3538, 415.1752, 387.1393, 383.1222, 355.1165, 327.1256	0.049
64	Hymenain	16.70	C_20_H_14_O_8_	[M+H]^+^	383.0761	383.0762	0.26	351.2722, 267.2624, 241.2161, 201.1194, 158.1122	0.017
65	Chimsalicifoliusin A	16.74	C_21_H_16_O_9_	[M+H]^+^	413.0867	413.0858	-2.18	413.4084, 395.4293, 385.3285, 367.6625, 275.2887, 227.5899	0.022
66	Arteminorin A	16.84	C_22_H_18_O_10_	[M+H]^+^	443.0973	443.0979	1.35	425.4505, 387.3369, 237.0622, 221.2556, 207.0823, 179.2379	0.028
67	**4-Hydroxy-1,10-secocadin-5-ene-1,10-dione**	16.95	C_15_H_24_O_3_	[M+H]^+^	253.1798	253.1799	0.39	235.4263, 217.3340, 199.3908, 289.1205, 169.1463, 111.2576	0.641
				[M-H]-	251.1650	251.1658	3.19	233.1077, 207.0895, 205.1244, 189.1191, 153.0140, 111.2131, 95.2395	
68	**Kaempferol**	17.07	C_15_H_10_O_6_	[M+H]^+^	287.0550	287.0553	1.05	287.0768, 241.1896, 213.2784, 165.0745, 153.1658	19.285
69	Cucumin E	17.18	C_15_H_20_O_2_	[M+H]^+^	233.1537	233.1535	-0.86	215.2999, 187.2966, 177.3355, 147.2748, 134.9405	2.291
70	(3-Acetyl-6,7-dihydroxy-7-methyl-4-propan-2-yl-1, 2,3,3a,4,5,6,7a-octahydroinden-5-yl) acetate	17.47	C_17_H_28_O_5_	[M+H]^+^	313.2010	313.2007	-0.96	314.2581, 257.3080, 239.5158, 165.3440, 123.0718, 109.1681	0.034
71	Robinlin	17.79	C_11_H_18_O_3_	[M+H]^+^	199.1329	199.1333	2.01	183.1020, 181.1223, 167.1069, 164.0834	0.011
72	Bullatantriol/4-epi-Bullanatanriol(1*β*,4*α*,11-oppositanetriol)	18.53	C_15_H_28_O_3_	[M+H]^+^	257.2111	257.2104	-2.72	239.2010, 223.1692, 211.1699, 181.1230	0.003
73	Oplodiol	18.94	C_15_H_26_O_2_	[M+H]^+^	239.2006	239.2003	-1.25	221.3019, 203.2772, 175.3271, 163.2742, 143.2712	0.049
74	Oxyphyllenodiol A/B	19.40	C_14_H_22_O_3_	[M+H]^+^	239.1642	239.1640	-0.84	221.2058, 203.2982, 185.1899, 161.2310, 95.2554	0.538
75	(1R,3R,6S,7R,10S)-7-Isopropyl-4,10-dimethylbicyclo[4.4.0]-dec-4-ene-3,10-diol (Y., 2013)	19.82	C_14_H_24_O_2_	[M+H]^+^	225.1849	225.1850	0.44	207.2649, 189.2796, 267.2000, 147.1779	0.426
76	4-Eudesmene-1*β*,11-diol	20.31	C_15_H_26_O_2_	[M+H]^+^	239.2006	239.2002	-1.67	221.2938, 203.4146, 185.2233, 129.1857, 121.0872	0.019
77	2,6-Dihydroxyhumula-3(12),7(13), 9(*E*)-triene	20.98	C_14_H_24_O_2_	[M+H]^+^	225.1849	225.1844	-2.22	207.3901, 189.3300, 147.1490, 133.2743	0.011
78	Pyrocatechol	21.41	C_6_H_6_O_2_	[M+H]^+^	111.0441	111.0438	-2.70		0.021
79	***E*-4-(4,8-dimethylnona-3,7-dienyl)furan-2(5H)-one**	21.89	C_15_H_22_O_2_	[M+H]^+^	235.1693	235.1690	-1.28	217.1238, 189.2911, 175.2929, 165.0198, 139.1942	6.063
				[M-H]-	233.1550	233.1547	-1.29	233.1511, 217.0485, 204.9865, 189.1096, 134.7004	
80	Homalomenol A	22.12	C_15_H_26_O_2_	[M+H]^+^	239.2006	239.2002	-1.67	221.4045, 203.1988, 175.2854, 161.2711, 95.2178	0.033
81	(+)-Δ-Cadinene	22.61	C_15_H_24_	[M+H]^+^	205.1951	205.1949	-0.97	163.2395, 149.2708, 135.2421, 121.2355, 109.2559	0.147
82	Diisobutyl phalate	22.68	C_16_H_22_O_4_	[M+H]^+^	279.1591	279.1596	1.79	261.2416, 219.1435, 205.0251, 201.3139, 149.1031	0.047
83	Farnesyl acetate	22.88	C_17_H_28_O_2_	[M+H]^+^	265.2162	265.2163	0.38	247.3895, 233.3257, 221.4232, 205.3046, 189.3889	0.013

The targeted antimicrobial compounds were marked in bold; ^a^ RC (relative content) was calculated as the percentage of peak area.

### 3.2 Computer-assisted structure elucidation by MS

**Figure 2:**
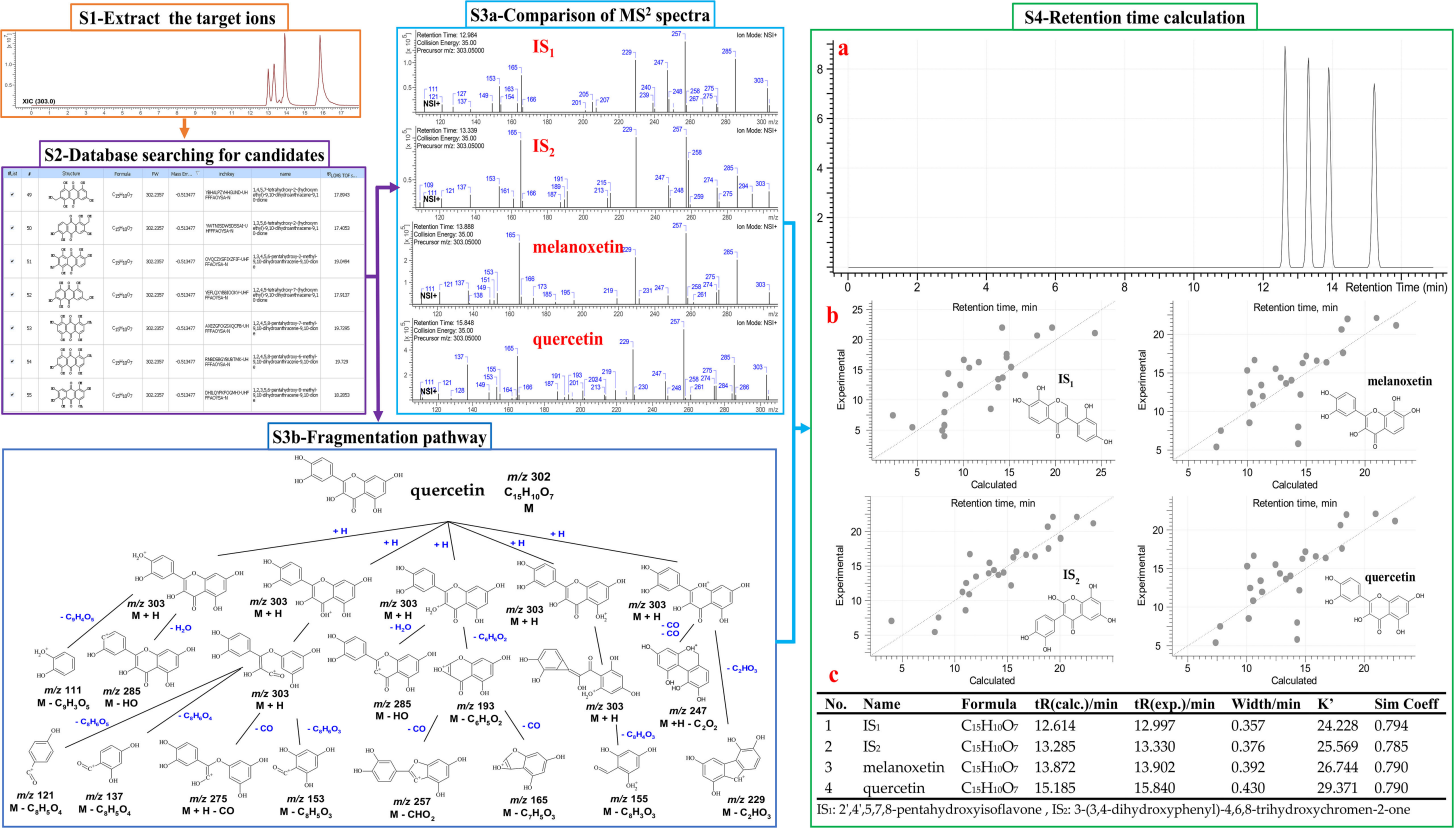
MS data analysis method. S1-4: identification steps 1-4. S1: the extracted ion chromatography XIC of 303.0499 m/z [C_15_H_11_O_7_] ^+^ in positive mode. S2: search with the exact mass in the COCONUT database. S3A: fragments of the four ions in MS^2^ spectra. S3B: the main fragmentation pathway of quercetin predicted by MS Fragmenter. S4: chromatographic retention time predictions **(a)**, regression curves **(b)** of four isomers by ChromGenius, and the calculated parameters **(c)**.

UHPLC-HRMS-MS data was analyzed for chemical identification in several steps. Firstly, precursor ions were obtained by auto calculation in the ACD/MS Workbook Suite. Then, the ultra-high-resolution mass was searched in chemical databases, such as COCONUT (https://coconut.naturalproducts.net/), and a manually built database of *Chimonanthus* plants. The latter (custom) database (**Supplementary Material 1**) should provide the best chance for correct identifications, because these compounds were isolated and identified from *Chimonanthus* plants before. In all, 83 compounds were identified from the ChG extract, including 17 isomers of 39 compounds. To illustrate the details, taking [M+H]^+^ 303.0499 *m/z* and [M-H]^-^ 301.0350 *m/z* precursor ions as an example, there were 4 peaks from the ChG extract with the same ions eluting at the retention times of 12.997, 13.330, 13.902, and 15.840 min (**Figure 2**-S1). The mass was compatible with the chemical formulas C_15_H_10_O_7_ or C_15_H_11_O_7_^+^. After searching the ultra-high-resolution mass in databases, 55 isomers were found (**Figure 2**-S2), of which only quercetin was reported in *Chimonanthus* plants before (Shu et al., 2019). Therefore, public databases were searched for fragmentation spectra corresponding to the experimental MS^2^ spectra(**Figure 2**-S3a). In addition, MS Fragmenter was used to predict the fragmentation patterns for the compounds without available spectra (**Figure 2**-S3b). Those four isomers have quite similar fragments in MS^2^ spectra, suggesting that their structures could come from the same chemical class. In this step, most of the 55 isomers were eliminated, and several possible ones were retained as candidates according to the fragmentation pattern predictions. The main fragmentation pathway of quercetin is shown in **Figure 2**-S3B, in which the most intense peaks were assigned by MS Fragmenter, such as 257 (C_14_H_11_O_6_^+^), 229 (C_13_H_9_O_4_^+^), 165 (C_8_H_5_O_4_^+^), 137 (C_7_H_5_O_3_^+^), 285 (C_15_H_9_O_6_^+^), 303 (C_15_H_11_O_7_^+^), 247 (C_13_H_11_O_5_^+^), 111 (C_6_H_7_O_2_^+^), 121 (C_7_H_5_O_3_^+^), 275 (C_14_H_11_O_6_^+^), 193 (C_9_H_5_O_5_^+^), etc. To confirm these predictions and also distinguish the isomers with similar structures, ChromGenius was used for retention time calculations (**Figure 2**-S4), then those four isomers were assigned as the final identifications.

Based on those steps, the spectra of all four compounds can be interpreted and verified: 2’,4’,5,7,8-pentahydroxyisoflavone (IS_1_), 3-(3,4-dihydroxyphenyl)-2,6,8-trihydroxy-4H-chromen-4-one (IS_2_), melanoxetin, and quercetin. Those four compounds come from two core structures, isoflavone (IS_1_, IS_2_) and flavones (melanoxetin, quercetin) substituted with hydroxyl groups at different positions. This leads to different polarity notwithstanding chemical similarity (Willett et al., 1998), so they can be distinguished *via* ChromGenius for retention time prediction. The calculation of retention time was performed under the same UHPLC-HRMS-MS condition for all 55 isomers. The chromatography of the final four isomers was generated by the calculation with the regression equation: 0.03*tR^2^ + 1.936*tR - 6.696, and are listed in **Figure 2**-S4c, where these four components gave k’ values (retention time factor) and similarity coefficients (Sim Coeff) as 24.228, 25.569, 26.744, 29.371, and 0.794, 0.785, 0.790, 0.790, respectively. The k’ values measured the period of time that the sample component resided in a stationary phase relative to the time it resides in the mobile phase, and it was calculated as (tR – t0)/t0, in which tR and t0 implied the retention time and dead time, respectively. According to the definition in ChromGenius, Sim Coeff is applied by the structural similarity to the current, and the value which stands closer to 1 provides better results for the calculation. Hence, it was considered that the calculation of these four isomers in **Figure 2**-S4 exhibited sufficient reliability. Moreover, to check the mass identification and computer-assistant elucidation method (McEachran et al., 2018; Hu et al., 2021a), a reference compound (quercetin) was measured under the same condition, and the retention time and fragmentation pattern were used to confirm the predicted results.

Another 16 sets of isomers were similarly analyzed based on their calculated tR: isomers in positive mode of *m/z* 149.0597 (C_9_H_8_O_2_), 153.0546 (C_8_H_8_O_3_), 193.0495 (C_10_H_8_O_4_), 209.0444 (C_10_H_8_O_5_), 225.1849 (C_14_H_24_O_2_), 237.0757 (C_12_H_12_O_5_), 239.2006 (C_15_H_26_O_2_), 287.0550 (C_15_H_10_O_6_), 347.2230 (C_22_H_26_N_4_), 355.1024 (C_16_H_18_O_9_), 383.0761 (C_20_H_14_O_8_), 385.1129 (C_17_H_20_O_10_), 387.1074 (C_20_H_18_O_8_), 443.0973 (C_22_H_18_O_10_), 449.1078 (C_21_H_21_O_11_^+^ or C_21_H_20_O_11_), and 595.1657 (C_27_H_31_O_15_^+^ or C_27_H_30_O_15_). Although the MS with the computer-assisted method is excellent to distinguish most isomers, there are still 2 types of isomers not identified due to their quite similar structures which require NMR identification for the pure compounds, such as *m/z* 347.2230 (C_22_H_26_N_4_) and *m/z* 443.0973 (C_22_H_18_O_10_). For *m/z* 347.2230, there are two isomer peaks in the ChG extract, where the first peak corresponds to either (+)-calycanthine, (-)-calycanthine, or (-)iso-calycanthine, and the second peak is meso-chimonanthine or chimonanthine. The two peaks of *m/z* 443.0973 were identified as 4,4’-biisofraxidin (or 3,3’-biisofraxidin), and arteminorin A, respectively.

The fragmentation prediction and retention time calculation provided additional information to narrow the scope of chemical candidates and have been used by many researchers before(Pelander et al., 2009; Blaženović et al., 2018; McEachran et al., 2018; Tabudravu et al., 2019). However, the method could be doubted, especially for the reliability of the compound fragmentation patterns, which can be influenced by experimental factors, such as instrument type, dissociation technique, ionization mode, collision energy, etc. According to previous studies, the fragmentation mechanisms are highly reproducible even across instruments (Stein, 2012; Hufsky et al., 2014), and the variations were mainly from the intensity of the fragments. Hence, it is feasible to do the comparison based on the predicted fragments (Champarnaud and Hopley, 2011). In MS Fragmenter, the identification scores for comparing the experimental fragmentation with the predictions were ranked, but it is clear that if we only focus on the scores generated by software, the fragmentation predictions will not be reliable for chemical identification (Schymanski et al., 2009). Therefore, we also used ChromGenius to check the retention time of all the chemical candidates. Afterward, the final results were generated according to both of them.

### 3.3 Antimicrobial activities of *C. grammatus* extracts

**Figure 3:**
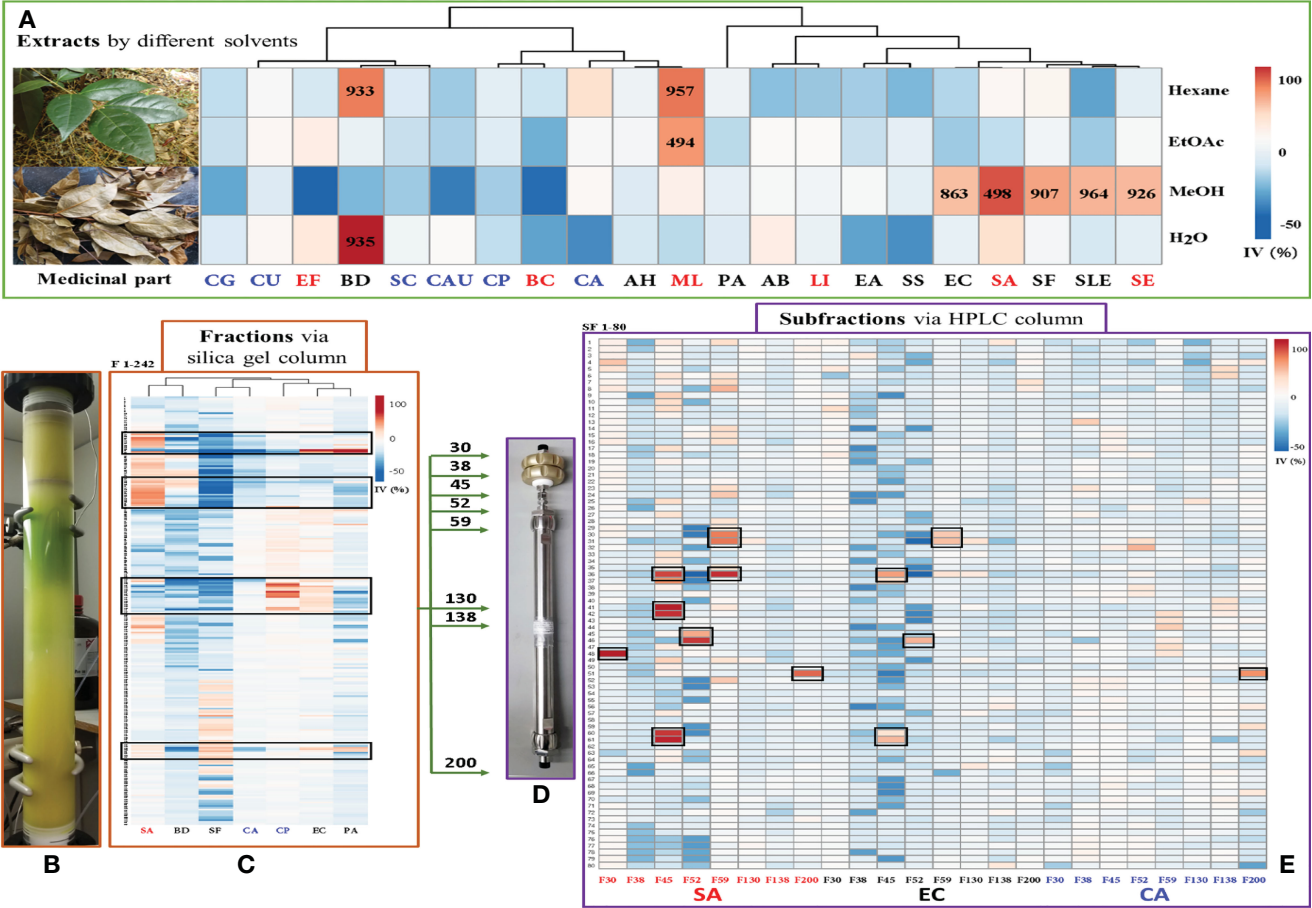
Bioassay-guided purification work of antimicrobial compounds in *C grammatus*. **(A)** Image of plant materials and the heat map of antimicrobial activities (IC_50_ in μg/mL) of different extracts; **(B)** Normal-phase (silica gel) column; **(C)** Heat map of antimicrobial activity of chromatographic fractions; **(D)** reversed-phase (C18) column; **(E)** Heat map of activity of subfractions against SA, EC, and CA, as representatives of G^+^, G^–^ bacteria, and fungi. IV: inhibition value; The microbes’ abbreviations are six fungi, nine G^–^ bacteria, and six G^+^ bacteria marked in blue, black, and red, respectively.

Four solvents with different polarities (water, methanol, ethyl acetate, and hexane), were used to extract the stem and leaf powder of ChG. The dried extracts, redissolved in DMSO, were tested against 21 different microbes, and the antimicrobial activities were shown in **Figure 3A**. ChG extracts inhibited the growth of three G^+^ and four G^–^ bacteria (IV>50%), and their IC_50_ values are lower than 1 mg/mL estimated from a two-fold dilution series. So far, other bioactivities of different ChG parts have also been reported: antioxidant activity (IC_50 _= 0.5-10 mg/mL by DPPH assay) of flower extracts due to the flavonoids (Zhang et al., 2017), antioxidant (IC_50 _= 12.145 mg/mL by DPPH assay) and antimicrobial activity of leaf essential oil (MIC = 2.25-4.5 mg/mL against *E. coli*, *P. aeruginosa*, *S. aureus*, *S. typhi*, *S. dysenteriae*, *B. subtilis*, *B. thuringiensis*, *S. luteus*, and *A. aerogenes* (Liu et al., 2011), AChE activity (IC_50_ = 8.153 and 4.812 mg/mL for petroleum ether and n-BuOH extract, respectively) and cytotoxicity of leaf extracts (0.5-1 mg/mL against A549 cells) (Ling, 2014). Here, the hexane extract of ChG showed activity against *B. diminuta* and *M. luteus* (IC_50 _= 933 and 957 μg/mL, respectively), and the EtOAc extract exhibited good inhibition against *M. luteus* (IC_50 _= 497 μg/mL). The MeOH extract had broad spectrum against five bacteria, including two G^+^ bacteria (*Staphylococcus epidermidis* and *S. aureus*, IC_50 _= 926 and 498 μg/mL, respectively), and three G^–^ bacteria (*Escherichia coli*, *S. flexneri* and *S. enterica* subsp. *enterica*, IC_50 _= 863, 907 and 964 μg/mL, respectively), while the water extract only inhibited the growth of *B. diminuta* (IC_50 _= 935 μg/mL). The MeOH and EtOAc extracts showed broad spectrum with high inhibition values, thus, they were used to extract ChG for bioassay-guided purification to identify the bioactive compounds.

In *Chimonanthus* genus, there are five other species, including *C. praecox*, *C. salicifolius*, *C. nitens*, *C. zhejiangensis*, and *C. campanulatus*. Previous studies mainly concentrated on the first three species, which extracts had activity on antimicrobial, anti-inflammatory, anti-tumor, analgesic, antitussive, expectorant, antipyretic, etc. (Shu et al., 2019) By now, the antimicrobial activities have been reported for the extracts of *C. nitens* leaves, *C. salicifolius* leaves, *C. zhejiangensis* leaves, *C. praecox* seed, as well as the essential oil of *C. praecox* leaves*, C. grammatus* leaves, and the total flavonoids of *C. salicifolius* leaves. The methanol extract of *C. praecox* seed can inhibit several plant pathogens, such as *Fusarium oxysporum*, *Sclerotinia sderotiorum*, *Alternaria solani*, *Setosphaeria turcica*, and *Bipolaris maydis* (Jiwen et al., 2005). The water extract of *C. nitens* leaves was active against *S. aureus*, *E. coli*, *P. aeruginosa*, *S. flexneri*, *Diplococcus pneumoniae*, *Hemolytic Streptococcus* B, and *Influenza Bacillus* (Ping et al., 1996; JC et al., 2002). The ethyl acetate, dichloromethane, and n-hexane fractions from the methanol extract of *C. salicifolius* leaves were reported to inhibit *S. aureus*, *E. coli*, *Bacillus subtilis*, and *P. aeruginosa* (Yongxiang et al., 2017). The water and ethanol extracts of *C. zhejiangensis* can inhibit *S. aureus*, *E. coli*, *P. aeruginosa*, *Klebsiella aerogenes*, *Salmonella typhi*, *K. pneumoniae*, *S. pneumoniae*, *Haemophilus influenzae* (Dongqing et al., 2005). Moreover, the essential oil of *C. praecox* leaves inhibited *S. aureus*, *B. subtilis*, and *Proteus* species (Zhuxiang and Gongxi, 2010), and the essential oil of *C. praecox* root and fruits were active against *S. aureus* and *C. albicans* (Zhifen et al., 2019), while *C. praecox* flower esential oil inhibited *S. epidermidis*, *E. coli*, *Bacillus coagulans*, *Sarcina luteu*, *S. aureus*, *B. subtilis*, *K. pneumoniae*, *Proteus vulgaris*, *S. epidermidis* (Ying et al., 2010). The total flavonoids of *C. salicifolius* leaves exhibited activity against *S. typhimurium*, *S. enteritidis*, *E. sakazakii*, *S. paratyphi* B, *S. castellani*. *E. coli* and *B. cereus* (Huiping et al., 2018). Compared with other *Chimonanthus* plants, the different ChG extracts showed the antimicrobial activity against several pathogens in this study, supporting its potential as a medicinal herb.

### 3.4 Active compounds elucidated *via* bioassay-guided purification

**Figure 4:**
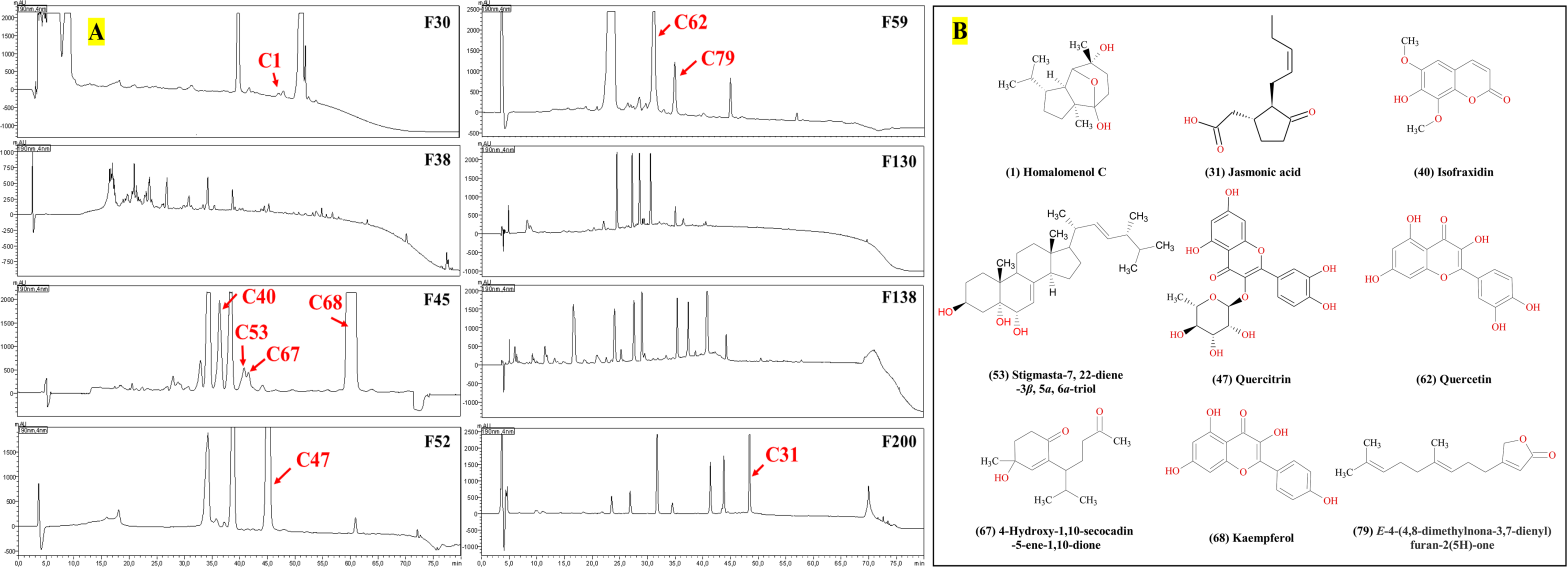
Antimicrobial compounds in the HPLC–DAD chromatograms **(A)** from *C. grammatus*, and their structures **(B)**.

A total of 6.4 g ChG extracts were obtained from the plant materials, then resolved on a normal-phase Silica gel column (**Figure 3B**) into 242 fractions. Several microorganisms were tested with each fraction, and the inhibition values of all 242 fractions were clustered in a heat map (**Figure 3C**). The eight most active fractions (F30, 38, 45, 52, 59, 130, 138, and 200) were selected for further purification. Afterward, around 2 mg of these fractions were injected into HPLC with a semipreparative reversed phase C18 column for isolating the active compounds. From each HPLC run, 80 subfractions were collected after optimizing separation conditions (see **Table 1**) and tested against selected microorganisms: *S. aureus* (Gram-positive), *E. coli* (Gram-negative), and *C. albicans* (fungus), which were amongst the most prevalent causes of bacterial and fungal infections in humans. The inhibition values of each subfraction were shown in **Figure 3D**, and their HPLC-DAD chromatographs in **Figure 4A**. Thus, 12, 6, and 1 active subfractions were obtained and assigned in the peaks of chromatographs. These peaks were collected and retested for their antimicrobial effects to confirm their activity, then they were analyzed by UHPLC-HRMS-MS for chemical identification.

The active peaks were analyzed in both positive and negative ion mode by the mass spectrometer, showing that all 13 peaks corresponded to nine different compounds, homalomenol C (1), isofraxidin (40), 4-hydroxy-1,10-secocadin-5-ene-1,10-dione (67), stigmasta-7,22-diene-3β,5α,6α-triol (53), kaempferol (68), E-4-(4,8-dimethylnona-3,7-dienyl)furan-2(5H)-one (79), quercitrin (47), jasmonic acid (31), and quercetin (62) (for their identification, see 3.2). All these compounds were already isolated from *Chimonanthus* plants, which supports their identification. Their structures were shown in **Figure 4B**. According to the bioassay-guided purification, all nine compounds inhibited the growth of the Gram-positive bacterium, *S. aureus*, while compounds 40, 68, and 62 were also active against *E. coli*, and only compound 31 was active against *C. albicans* (**Figures 3E**, **4A**).

### 3.5 Antimicrobial and antibiofilm assessments against *S. aureus*

**Figure 5:**
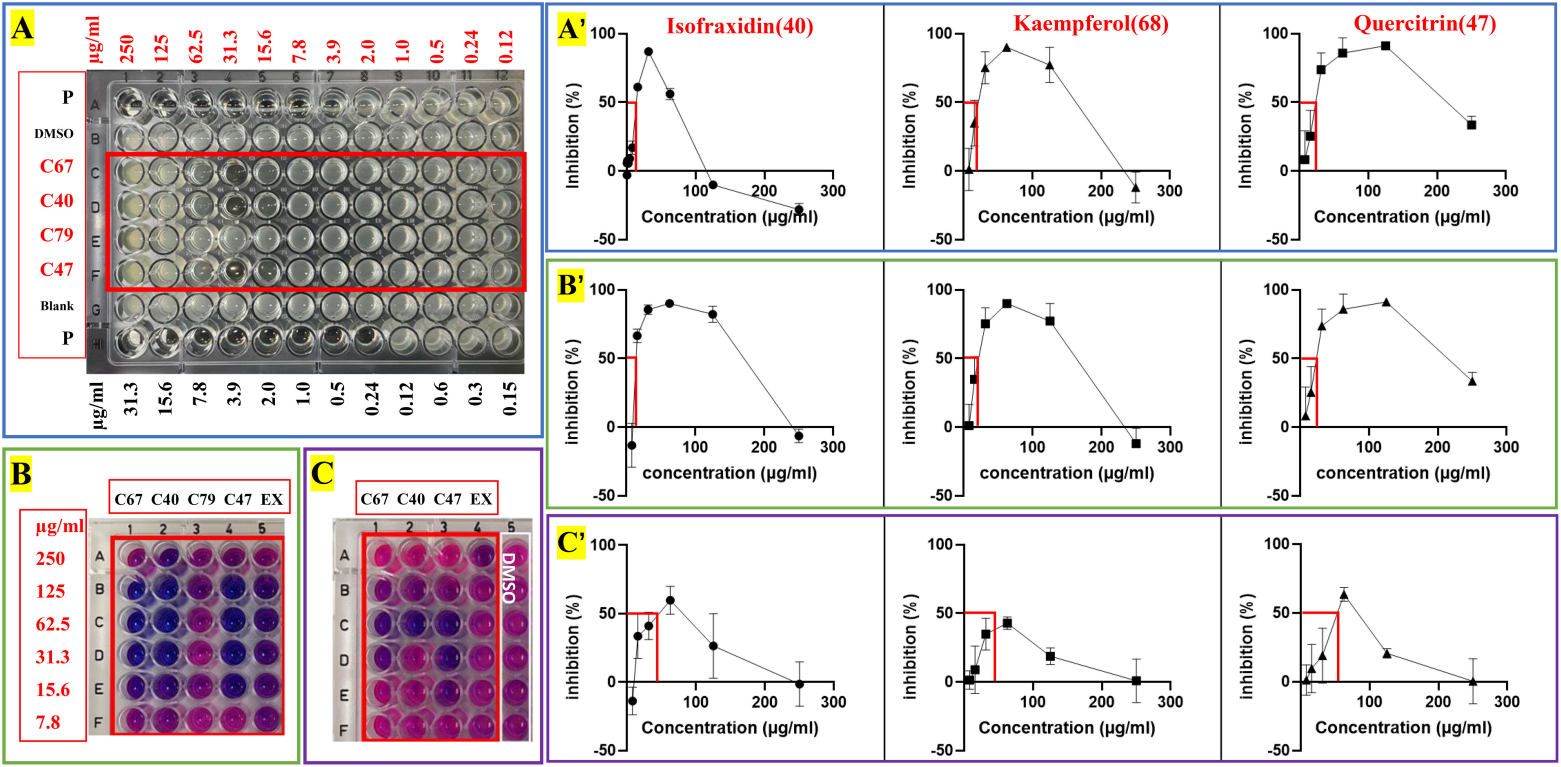
Antimicrobial and anti-biofilm activity against *S. aureus* of crude extracts and four pure compounds from *C grammatus*. **(A)** IC_50_ test; **(B)** biofilm formation test; **(C)** preformed biofilm test; P, Ciprofloxacin; EX, ChG extract (two-fold serial dilutions from 1000 µg/mL).

For the active compounds, only four of them can be collected for more than 1 mg in this study due to the method limitation, which we used to do the further activity tests, including 2.6 mg isofraxidin (40), 5.3 mg kaempferol (68), 2.8 mg quercitrin (47), 1.1 mg *E*-4-(4,8-dimethylnona-3,7-dienyl)furan-2(5H)-one (79). They were evaluated against two strains of *S. aureus* (ATCC6538 and USA300) for their potentials as antimicrobial reagents, including IC_50_ and MBC for antimicrobial activity, as well as BIC_50_ and BEC_50_ for anti-biofilm activity. For MBC, colony-forming units (CFU) were calculated to determine if the bactericidal effect was greater than 99%. All these tests were done by two-fold serial dilutions in DMSO with concentrations ranging from 0.12-250 µg/ml. The positive controls were ciprofloxacin and sodium dodecyl sulfate (SDS), which were diluted as 0.15-31.3 µg/ml and 0.02%-20%, respectively. The inhibition curves were analyzed by GraphPad and inferred parameters were listed in **Table 3** and **Figure 5**.

**Table 3:** Antimicrobial and anti-biofilm activity against *S. aureus*.

Activity	Methanol extract (µg/mL)	Isolated compounds (µg/mL)				Positive control	
		Isofraxidin (40)	Kaempferol (68)	C_79_^*^	Quercitrin (47)	Ciprofloxacin (µg/mL)	SDS (%)
**IC_50_**	498	13.51	18.08	> 250	15.86	0.16	NT
**MBC**	> 2000	> 250	> 250	> 250	> 250	≥ 1.25	NT
**BIC_50_**	> 1000	15.43	17.31	> 250	18.86	> 31.30	< 0.625%
**BEC_50_**	> 1000	45.86	≥ 62.50	> 250	57.62	> 31.30	< 0.625%

^*^ C_79_: E-4-(4,8-dimethylnona-3,7-dienyl)furan-2(5H)-one

Here we noticed that higher concentrations (250, 125 µg/ml) of compounds 40, 68, and 79 showed lower inhibition values than the low concentrations (62.50, 31.30, 15.65 µg/ml) according to the OD and fluorescence measurements (**Figures 5A–C**). A similar phenomenon of kaempferol was reported against *Bacillus cereus*, which showed strong inhibition with ≥14mm inhibition zone in 10 μM (2.9 mg/mL) while 5 and 20 μM (1.4 and 5.7 mg/mL) had moderate activity with a 12-13.9 mm inhibition zone (Lee et al., 2011). The same was also observed for nalidixic acid against *E.coli*, where increasing the concentration after a certain point showed decreased bactericidal activity (Crumplin and Smith, 1975). Nalidixic acid at low concentrations can cause single-strand nicks in DNA, leading to DNA degradation and bacterial death, while higher concentrations can also inhibit RNA and protein synthesis, which prevents the synthesis of nucleases and reduce the DNA nicks because nucleases are needed to generate DNA nicks. The fact that a compound is bacteriostatic in high concentrations and bactericidal in low concentrations has important clinical implications since these compounds should preferably be used at a suitable low concentration, which may also provide other advantages like less adverse effects and less resistance development. Moreover, this interesting observation deserve further research, especially given its relevance for their mechanism of action.

In this study, IC_50_, BIC_50_, and BEC_50_ were calculated by the front part of each curve (**Figures 5A–C**), which means the low inhibition values of these high concentrations for each compound were not included in these calculations. Based on this, the isolated compounds, isofraxidin (40), kaempferol (68), and quercitrin (47) showed significant activity against planktonic *S. aureus* (IC_50 _= 13.5, 18.08, and 15.86 µg/ml), while *E*-4-(4,8-dimethylnona-3,7-dienyl)furan-2(5H)-one (79) exhibited slight activity (IC_50_ > 250 µg/ml). Although the positive control, ciprofloxacin, has roughly a 100-fold lower IC_50_ value (0.16 µg/ml), the compounds 40, 47, and 68 can nonetheless be considered as potential antimicrobial reagents. Indeed the well-known natural product, berberine, showed similar IC_50_ values (14.6 µg/ml) (Cernakova and Košťálová, 2008), and has been widely used as a herbal antibiotic for treating diarrhea and other gastrointestinal disorders for several decades (Gaba et al., 2021). On the other hand, they showed much higher antibiofilm activity (BIC_50 _= 15.43, 17.31, 18.86 µg/ml) than ciprofloxacin, especially for inhibiting biofilm formation. Also, these three compounds (BEC_50 _= 45.86, ≥62.50, and 57.62 µg/ml) can eradicate a preformed biofilm of *S. aureus*.

### 3.6 Discussion on the bioactivity of ChG active compounds

All nine active compounds were already reported in *Chimonanthus* species. Here, we list their published bioactivities (**Table 4**) and compare those with our results. Homalomenol C (1) was already reported to have antiplasmodial activity, cytotoxicity, and antifungal activities, while this is the first report about its activity against *S. aureus*. Jasmonic acid (31) is a fatty acid distributed in higher plants, bacteria, and fungi. In addition to being a famous plant hormone, it also has multiple bioactivities, including anticancer, antidepressant, anti-aggressive, antimicrobial, antioxidant, anti-inflammatory, anti-nociceptive, antiparasitic, etc. It was identified as an antifungal component against *Pyricularia oryzae* (syn. *Magnaporthe oryzae*) from wild rice. Isofraxidin (40) exerts cytotoxic, anti-fatigue, anti-stress, cholagogic, anti-inflammatory effects, protection against acute lung injury in mice, antimalarial, analgesic, sedative, hypnotic, anti-complement, antitussive, antioxidant, anti-diabetic, antilipidemic, antiviral activity, and antimicrobial activity against *B. subtilis*, *S. aureus*, *E. coli*, *C. albicans*, *Aspergillus niger*, and *Ralstonia solanacearum*. Our study adds antibiofilm activity against *S. aureus*. Quercitrin (47) is a component commonly found in various plants. So far, many bioactivities have been documented, including antiinflammation, antioxidative stress, antimicrobial, immunomodulation, analgesia, wound healing, vasodilation, etc. Increasing evidence has implied its antimicrobial effect against various microbes (viruses, in addition to over 22 bacteria, and 12 fungi), including *S. aureus*, *E. coli*, *C. albicans*, etc. Stigmasta-7,22-diene-3β,5α,6α-triol (53), and 4-hydroxy-1,10-secocadin-5-ene-1,10-dione (67) were active against *S. aureus* in this study; this activity of compound 67 was reported before, while the antimicrobial activity of compound 53 has not been published before. So far, no other bioactivity has been reported for them; this will require further research. Quercetin (62) is also a common compound represented in various plants, which was first described in 1936 and widely used due to its various actions, including antioxidant, anticancer, anti-inflammatory, hepato-protective, antiallergic, and antimicrobial activities. Its wide antimicrobial spectrum has been proven against over 32 bacteria and nine fungi. In addition, it also shows anti-biofilm activity against *S. aureus*. Kaempferol (68) is a very common compound that has been found in many herbal medicines and edible plants. It gained popularity due to its pharmacological properties, including antioxidant, anti-inflammatory, antibacterial, antiviral, antifungal, antiprotozoal, anticancer, hepatoprotective, neuroprotective, and cardioprotective activity, etc. As an antimicrobial, kaempferol is active against over 37 bacteria and nine fungi, including *S. aureus*, *E. coli*, *C. albicans*, etc. Kaempferol was also found active against biofilms, including *S. aureus*, *C. parapsilosis*, etc. *E*-4-(4,8-dimethylnona-3,7-dienyl)furan-2(5H)-one (79) has been isolated from *Chimonanthus* species and was reported to be active against *B. subtilis*, and *E. coli*, as well as diisobutyl phalate and 4-hydroxy-3-[(2*E*,6*E*)-3,7,11-trimethyl-2,6,10-dodecatrien-1-yl] benzoic acid, while scopoletin only showed activity against *B. subtilis.* (Ya et al., 2013). However, this study didn’t show its activity against *E. coli* during bioassay-guided separation, which could be the reason for the low content.

**Table 4:** Bioactivities of nine compounds from *C. grammatus*.

Compound	Activities	IC50 (μg/ml)	MIC (μg/ml)	Inhibition zone (mm)	Literature
**C_1_:** Homalomenol C	antiplasmodial activity against *Plasmodium falciparum*	1.2	–	–	(Henchiri et al., 2009)
	none significant cytotoxicity against human diploid lung cell line MRC-5	> 100	–	–	
	cytotoxicity against human hepatocarcinoma SNU739 cancer cells and gastric carcinoma NUGC3	–	–	–	(Wang et al., 2011)
	moderate cytotoxicity against HL-60 cell lines	9.64	–	–	(Yang et al., 2016b)
	cytotoxicity against SMMC-7721 cell lines	12.06	–	–	
	cytotoxicity against HeLa cell lines	9.54	–	–	
	a stimulative effect on the proliferation and differentiation of culture osteoblasts	0.03 ~ 3			(Hu et al., 2008)
	**antifungal activity** against *Penicillium italicum*	14.37	–	–	(Yang et al., 2016b)
	**antifungal activity** against *Rhizoctonia solani*	12.54	–	–	
	none or slightly anti-inflammatory activity of NO-production inhibition in LPS-stimulated RAW 264.7 cells	24.95 ± 1.69	–	–	(Yang et al., 2019) (Thuy et al., 2022)
**C_40_:** Isofraxidin	inhibit matrix metalloproteinase-7 expression and cell Invasion of human hepatoma cells	–	–	–	(Yamazaki and Tokiwa, 2010)
	cytotoxicity against A549 human lung cancer cells	9.49±0.90 for 24 h treatment			(Zhang et al., 2018)
	cytotoxicity against BEAS-2B normal lung epithelial cells	18.96±0.52 for 48 h treatment			
	cytotoxicity against human colorectal cancer HT-29 cells	8.89	–	–	(Shen et al., 2017)
	cytotoxicity against human colorectal cancer SW-480 cells	17.78	–	–
	anti-fatigue, anti-stress	–	–	–	(Sun et al., 2007)
	cholagogic, anti-inflammatory effects	–	–	–	(Niu et al., 2012; Liu et al., 2015)
	acute lung injury protection in mice	–	–	–	(Niu et al., 2015)
	antimalarial activity against Plasmodium falciparum	7.95	–	–	(Cubukcu et al., 1990)
	analgesic effect	–	–	–	(Okuyama et al., 2001)
	sedative and hypnotic effects	–	–	–	(Kang and Li, 2016)
	anti-complement and antitussive activities	–	–	–	(Huang et al., 2020)
	antioxidant activity	–	–	–	(Wang et al., 2015)
	anti-diabetic, antili-pidemic effects	–	–	–	(Lu et al., 2022)
	**antiviral** activity against influenza A virus	250	–	–	(Wang et al., 2017)
	**antimicrobial** activity against *Bacillus subtilis*	–	37.5	–	(Hanmin, 2019)
	**antimicrobial** activity against *Escherichia coli*	–	75	–
	**antimicrobial** activity against *Staphylococcus aureus*	–	7 in 50 μg/ml		(Acharyya and Sarma, 2014)
	**antimicrobial** activity against *Candida albicans*	–	6.5 in 50 μg/ml	
	**antimicrobial** activity against *Aspergillus niger*	–	6.5 in 50 μg/ml	
	**antimicrobial** activity against *Ralstonia solanacearum*	–	–	–	(Yang et al., 2016a)
**C_67_**: 4-Hydroxy-1,10-secocadin-5-ene-1,10-dione	**antimicrobial** activity against *Staphylococcus aureus*	–	7 in 100 μg/disk		(Liu et al., 2017)
**C_53_**: Stigmasta-7,22-diene-3*β*,5*α*,6*α*-triol	none activity reported	–	–	–	(Li et al., 2016)
**C_68_:** Kaempferol	antioxidant, anti-inflammatory, antibacterial, antiviral, antifungal, antiprotozoal, anticancer, hepatoprotective, neuroprotective, and cardioprotective activity, etc.	–	–	–	(Calderon-Montano et al., 2011; Punia Bangar et al., 2022)
	**antimicrobial** activity against *Staphylococcus aureus*	–	>50	–	(Lin et al., 2020)
	**antimicrobial** activity against *Escherichia coli*	–	32	–	(Deng et al., 2021)
	**antimicrobial** activity against *Staphylococcus pneumoniae*	–	256	–	(Otsuka et al., 2008)
	**antimicrobial** activity against *Serratia marcescens*	–	>256	–
	**antimicrobial** activity against *Enterococcus faecium*	–	>256	–
	**antimicrobial** activity against *Enterococcus faecalis*	–	130.55 and 114.58 for different strains		(del Valle et al., 2016)
	**antimicrobial** activity against *Porphyromonas gingivalis*	–	20	–	(Cai and Wu, 1996)
	**antimicrobial** activity against *Prevotella intermedia*	–	20	–
	**antimicrobial** activity against *Streptococcus mutans*	–	2500	–
	**antimicrobial** activity against *Actinomyces viscosus*	–	1250	–
	**antimicrobial** activity against *Helicobacter pylori*	–	MBC=6	–	(Kataoka et al., 2001; Martini et al., 2009)
	**antimicrobial** activity against *Propionibacterium acnes*	–	≤32	–	(Lim et al., 2007)
	**antimicrobial** activity against *Bacillus subtilis*	–	–	1.33	(Sati et al., 2019)
	**antimicrobial** activity against *Micrococcus roseus*	–	–	1.00
	**antimicrobial** activity against *Pesudomanas putida*	–	–	1.37
	**antimicrobial** activity against *Fusarium oxysporum*	–	–	1.67
	**antimicrobial** activity against *Trametes hirsuta*	–	–	1.33
	**antimicrobial** activity against *Staphylococcus epidermidis*	–	>1024	–	(Siriwong et al., 2016)
	**antimicrobial** activity against *Bacillus cereus*	≥14 in 2.86 μg/ml			(Lee et al., 2011)
	**antimicrobial** activity against *Pseudomonas aeruginosa*	–	≥59	–	(Lee et al., 2014)
	**antimicrobial** activity against *Micrococcus luteus*	–	28.62	–	(Ulanowska et al., 2007)
	**antimicrobial** activity against *Sarcina sp.*	–	28.62	–
	**antimicrobial** activity against *Vibrio harveyi*	–	≤2.86	–
	**antimicrobial** activity against *Vibrio cholerae*	–	32-128	–	(Tatsimo et al., 2017)
	**antimicrobial** activity against *Shigella flexneri*	–	128	–
	**antimicrobial** activity against *Salmonella typhi*	–	–	10	(Aye et al., 2018)
	**antimicrobial** activity against *Shigella boydii*	–	–	10
	**antimicrobial** activity against *Plesiomonas shigelloides*	–	–	12
	**antimicrobial** activity against *Enterobacter aerogenes*	–	128 and 256 for different strains		(Kuete et al., 2011)
	**antimicrobial** activity against *Klebsiella pneumonia*	–	256	–
	**antimicrobial** activity against *Acinetobacter baumannii*	–	1000	–	(Rofeal et al., 2021)
	**antimicrobial** activity against fish spoilage bacteria (*Proteus mirabilis*, *Photobacterium damselae*, *Enterobacter cloacea*, *Serratia liquefaciens* and *Pseudomonas luteola*) and food-borne pathogens (*Yersinia enterocolitica, etc.*) via biogenic amine production test	–	–	–	(Özkütük, 2022)
	**antimicrobial** activity against *Candida albicans*	–	15	–	(Lee et al., 2012)
	**antimicrobial** activity against *Aspergillus niger*	–	15	–
	**antimicrobial** activity against *Candida parapsilosis*	–	32-128	–	(Rocha et al., 2019)
	**antimicrobial** activity against *Candida orthopsilosis*	–	64	–
	**antimicrobial** activity against *Candida metapsilosis*	–	32-64	–
	**antimicrobial** activity against *Candida krusei*	–	64	–
	**antimicrobial** activity against *Cryptococcus neoformans*	–	16	–	(Tatsimo et al., 2012)
	**antimicrobial** activity against *Candida glabrata*	–	31.2	–	(Salazar-Aranda et al., 2015)
	**antimicrobial** activity against *Candida tropicalis*	–	>83	–
	**antibiofilm** against *Streptococcus mutans*, *Staphylococcus aureus*, *Candida albicans*, *Candida parapsilosis*, *C. orthopsilosis*, and *C. metapsilosis.*	–	–	–	(Rocha et al., 2019; Zeng et al., 2019; Lan et al., 2020; Gao et al., 2021)
**C_79_:** *E*-4-(4,8-dimethylnona-3,7-dienyl)furan-2(5H)-one	**antimicrobial** activity against *Bacillus subtilis*	–	2000	–	(Ya et al., 2013)
	**antimicrobial** activity against *Escherichia coli*	–	1500	–
**C_47_:** Quercitrin	antiinflammation, antioxidative stress, antimicrobial, immunomodulation, analgesia, wound healing, vasodilation, etc. treating metabolic diseases, gastrointestinal diseases, cardiovascular, cerebrovascular diseases, etc.	–	–	–	(Chen et al., 2022)
	**antimicrobial** activity against *Escherichia coli*	–	100	–	(Ajibesin et al., 2011)
	**antimicrobial** activity against *Bacillus cereus*	–	50	–
	**antimicrobial** activity against *Staphylococcus aureus*	–	50	–
	**antimicrobial** activity against *Pseudomonas aeruginosa*	–	100	–
	**antimicrobial** activity against *Streptococcus mutans*	–	64	–	(Hasan et al., 2014)
	**antimicrobial** activity against *Propionibacterium acnes*	–	200	–	(Luan et al., 2019)
	**antimicrobial** activity against *Serratia marcescens*	–	1024	–	(Dong et al., 2016)
	**antimicrobial** activity against *Staphylococcus epidermidis*	<0.45		–	(Gómez-Florit et al., 2014)
	**antimicrobial** activity against *Vibrio anguillarum*	–	>2000	–	(Jang et al., 2018)
	**antimicrobial** activity against *Edwardsiella tarda*	–	2000	–
	**antimicrobial** activity against *Streptococcus iniae*	–	2000	–
	**antimicrobial** activity against *Salmonella typhimurium*	≈8	–	–	(Li et al., 2021)
	**antimicrobial** activity against *Salmonella enteritidis*	–	800	–	(Arima et al., 2002)
	**antimicrobial** activity against *Klebsiella pneumonia*	>256		–	(Lin et al., 2005)
	**antimicrobial** activity against *Bacillus subtilis*	–	200	–	(Gehrke et al., 2013)
	**antimicrobial** activity against *Staphylococcus epidermid*	–	200	–
	**antimicrobial** activity against *Streptococcus pyogen*	–	200	–
	**antimicrobial** activity against *Staphylococcus saprophyti*	–	100	–
	**antimicrobial** activity against *Shigella sonnei*	–	100	–
	**antimicrobial** activity against *Cryptococcus neoformans*	–	100	–
	**antimicrobial** activity against *Candida tropicalis*	–	100	–
	**antimicrobial** activity against *Saccharomyces cerevisiae*	–	200	–
	**antimicrobial** activity against *Listeria monocytogenes*	–	150	–	(Elansary et al., 2020)
	**antimicrobial** activity against *Mariniluteicoccus flavus*	–	130	–
	**antimicrobial** activity against *Aspergillus flavus*	–	170	–
	**antimicrobial** activity against *Aspergillus ochraceus*	–	180	–
	**antimicrobial** activity against *Aspergillus niger*	–	120	–
	**antimicrobial** activity against *Candida albicans*	–	270	–
	**antimicrobial** activity against *Penicillium funiculosum*	–	270	–
	**antimicrobial** activity against *Penicillium ochrochloron*	–	200	–
	**antimicrobial** activity against *Candida glabrata*	–	7.8-62.5	–	(Salazar-Aranda et al., 2015)
	**antimicrobial** activity against *Candida tropicalis*	–	>83	–
	**antimicrobial** activity against *Helminthosporium sativum*	–	50	–	(Lu et al., 2002)
**C_31_:** Jasmonic acid	anticancer, antidepressant, anti-aggressive, antimicrobial, antioxidant, anti-inflammatory, anti-nociceptive, antiparasitic, etc.	–	–	–	(Pirbalouti et al., 2014)
	**antifungal** activity against *Pyricularia oryzae* (syn. *Magnaporthe oryzae*) from wild rice	–	250	–	(Neto et al., 1991)
**C_62_:** Quercetin	antioxidant, anticancer, anti‐inflammatory, antioxidant, hepato‐protective, antiallergic, and antimicrobial activities, etc., preventing various diseases, e.g., osteoporosis, some forms of cancer, tumors, lung and cardiovascular diseases, even against aging, etc.	–	–	–	(Patel et al., 2018) (Rauf et al., 2018; Xu et al., 2019)
	**antimicrobial** activity against *Staphylococcus aureus*	–	33.8	–	(Lin et al., 2020)
	**antimicrobial** activity against *Escherichia coli*	–	77.4	–	(Alvarez et al., 2008)
	**antimicrobial** activity against *Streptococcus mutans*	–	2000	–	(Shu et al., 2011)
	**antimicrobial** activity against *Streptococcus sobrinus*	–	1000	–
	**antimicrobial** activity against *Streptococcus sanguis*	–	2000	–
	**antimicrobial** activity against *Lactobacillus acidophilu*	–	2000	–
	**antimicrobial** activity against *Porphyromonas gingivalis*	–	4000	–
	**antimicrobial** activity against *Aggregatibacter actinomycetemcomitans*	–	2000	–
	**antimicrobial** activity against *Helicobacter pylori*	–	10 in 100 mg/disk		(Ramos et al., 2006)
	**antimicrobial** activity against *Proteus vulgaris*	–	300	–	(Jaisinghani, 2017)
	**antimicrobial** activity against *Salmonella enterica* serotype Typhimurium	–	2.18	–	(Wang et al., 2018)
	**antimicrobial** activity against *Micrococcus roseus*	–	–	2.33	(Sati et al., 2019)
	**antimicrobial** activity against *Pesudomanas putida*	–	–	1.67
	**antimicrobial** activity against *Serratia marcescens*	–	–	2.00
	**antimicrobial** activity against *Fusarium oxysporum*	–	–	2.33
	**antimicrobial** activity against *Trametes hirsuta*	–	–	1.00
	**antimicrobial** activity against *Propionibacterium acnes*	–	≤64	–	(Lim et al., 2007; Lee et al., 2014)
	**antimicrobial** activity against *Salmonella enteritidis*	–	250	–	(Arima et al., 2002)
	**antimicrobial** activity against *Bacillus cereus*	–	350	–
	**antimicrobial** activity against *Salmonella typhi*	–	–	12	(Aye et al., 2018)
	**antimicrobial** activity against *Shigella boydii*	–	–	12
	**antimicrobial** activity against *Plesiomonas shigelloides*	–	–	14
	**antimicrobial** activity against *Enterobacter aerogenes*	–	128	–	(Kuete et al., 2011)
	**antimicrobial** activity against *Klebsiella pneumonia*	–	256	–
	**antimicrobial** activity against *Bacillus subtilis*	–	100	–	(Gehrke et al., 2013)
	**antimicrobial** activity against *Staphylococcus epidermid*	–	100	–
	**antimicrobial** activity against *Streptococcus pyogen*	–	100	–
	**antimicrobial** activity against *Staphylococcus saprophyti*	–	100	–
	**antimicrobial** activity against *Escherichia coli*	–	100	–
	**antimicrobial** activity against *Pseudomonas aeruginosa*	–	100	–
	**antimicrobial** activity against *Shigella sonnei*	–	100	–
	**antimicrobial** activity against *Micrococcus kristinae*	–	2500	–	(Abdel-Raouf et al., 2011)
	**antimicrobial** activity against *Sarcina maxima*	–	2500	–
	**antimicrobial** activity against *Helminthosporium sativum*	–	50	–	(LLu et al., 2002)
	**antimicrobial** activity against *Candida albicans*	–	100	–	(Gehrke et al., 2013)
	**antimicrobial** activity against *Cryptococcus neoformans*	–	100	–
	**antimicrobial** activity against *Candida tropicalis*	–	100	–
	**antimicrobial** activity against *Saccharomyces cerevisiae*	–	100	–
	**antimicrobial** activity against *Candida parapsilosis*	–	0.5-4	–	(Rocha et al., 2019)
	**antimicrobial** activity against *Candida orthopsilosis*	–	2-8	–
	**antimicrobial** activity against *Candida metapsilosis*	–	0.5-16	–
	**antimicrobial** activity against *Candida krusei*	–	2	–
	**antibiofilm** against Bacillus subtilis, *Enterococcus faecalis, E. faecium, Listeria monocytogenes, Staphylococcus aureus, Staphylococcus saprophyticus, Streptococcus mutans, Streptococcus pneumoniae, Candida parapsilosis, Candida orthopsilosis, Candida metapsilosis, Candida krusei* etc.	–	–	–	(Memariani et al., 2019; Rocha et al., 2019)

According to the previous study, there are many other compounds with antimicrobial activities isolated from *Chimonanthus* plants, including the alkaloids (calycanthine against plant pathogens, such as *Alternaria brassicicola*, *Cladosporium fulvum* and *Botrytis cinerea*; *d*-calycanthine and *l*-folicanthine against *Exserohilum turcicum*, *Bipolaris maydis*, *Alternaria solani*, *Sclerotinia sderotiorum*, and *Fusarium oxysportium*; etc.), the coumarins (scopoletin against *M. lutus* and *E.coli*; 5,6,7-trimethoxycoumarin, calycanthoside, and xeroboside against *M. lutus*; etc.) (Shu et al., 2019), the flavonoids, terpenoids, essential oil, etc. The chemical investigations in *Chimonanthus* revealed that these plants have high content of flavonoids (such as quercetin, kaempferol, rutin, hyperin, isoquercitrin, afzelin, etc.), coumarins and terpenoids [such as 21.43% 3-(4,8-dimethylnona-3,7-dienyl)-furan, 10.51% longifolenaldehyde, 11.85% (+)-2-bornanone, 5.69% Δ-cadinene of *Chimonanthus nitens*], while with low yield of bioactive alkaloids (Shu et al., 2019; He et al., 2022; Chen et al., 2023). The results of this study matched well with the chemical investigations of *Chimonanthus* plants, and the nine active compounds isolated from C. *grammatus* included three flavonoids (47, 62, 68), three terpenoids (1, 67, 79), one coumarin (40), steroid (53) and organic acid (31). These nine compounds have been reported in other *Chimonanthus* plants, such as homalomenol C from *C. praecox* fruit (Wang et al., 2011); Jasmonic acid from *C. praecox* flower bud (Zhang and Chen, 2012); isofraxidin from *C. praecox* flower bud, root, stem, *C. salicifolius* aerial parts and *C. nitens* leaf; quercitrin from *C. praecox* flower (Shu et al., 2019); stigmasta-7,22-diene-3β,5α,6α-triol from *C. salicifolius* aerial parts (Li et al., 2016); 4-hydroxy-1,10-secocadin-5-ene-1,10-dione from *C. praecox* rhizome (Y., 2013); quercetin from flower and leaf of *C. praecox*, *C. salicifolius*, *C. nitens*, *C. zhejiangensis*, and *C. grammatus*; kaempferol from flower, leaf in *C. praecox*, *C. salicifolius*, *C. nitens*, *C. zhejiangensis*, and *C. grammatus*; *E*-4-(4,8-dimethylnona-3,7-dienyl)furan-2(5H)-one from *C. grammatus* leaf (Wang, 2012; Shu et al., 2019).

However, many natural compounds were not very clear for their bioactivities, such as antimicrobial activities. Hence, it is worth doing the biotest even if they are known. The bioassay-guided purification work normally obtains the main active compounds from plant extracts, as well as their activities, which can be quite useful to know the chemical basis of medicinal herb’s bioactivities. Although these nine compounds isolated here are known, it is still important to figure out all of them from *C. grammatus via* bioassay-guided purification. These active compounds can be used for quality control when developing the plant as medicine. Furthermore, we report for the first time the activity of compounds 1, 53, and 79 against *S. aureus*, and the antibiofilm activity of compounds 40 and 79. The BIC_50_ and BEC_50_ of compounds 40, 47, and 68 were measured for the first time *via* a resazurin-based viability staining method for microbial biofilms. According to the MS semiquantitative analysis, compounds 68 (19.3% content), 79 (6.1%), 47 (4.0%), 62 (3.7%), and 40 (3.1%) dominated in the ChG extract, which are the main roles in the antimicrobial activities of ChG, according to the bioassay in this study. Kaempferol (68) most likely accounts for much of the antimicrobial activity of the ChG extracts, but its oral bioavailability is limited and its pharmacokinetics are unfavorable (rapid elimination and metabolism) (Barve et al., 2009; Zabela et al., 2016). Bioactive analogs or certain formulations may circumvent these limitations (Ren et al., 2019), and topical use may be possible (Özay et al., 2019). Its safety permits use in food products as a preservative (Martinengo et al., 2021). In this study, some minor compounds also showed antimicrobial activity, suggesting that it is worth pursuing even those by bioassay-guided purification. To summarize, all nine compounds could contribute to the antimicrobial activity of ChG extracts and their potential additive, synergistic or antagonistic effects remain to be established.

## 4 Conclusions

We showed activity of different *C. grammatus* extracts against SA (IC_50 _= 498 μg/mL for methanol extract), ML (IC_50 _= 957 μg/mL for hexane extract, 494 μg/mL for EtOAc extract), BD (IC_50 _= 933 μg/mL for hexane extract, 935 μg/mL for water extract), EC (IC_50 _= 863 μg/mL for methanol extract), SF (IC_50 _= 907 μg/mL for methanol extract), SLE (IC_50 _= 964 μg/mL for methanol extract), and SE (IC_50 _= 926 μg/mL for methanol extract). These different extracts are active against different microorganisms, which may suggest that the plant contains different antimicrobial compounds, presumably with different mechanisms of action. *Via* bioassay-guided purification work, nine active compounds were identified, and all are active against *S. aureus*, meanwhile compounds 40, 47, 62, and 68 are active against *E. coli*, and only compound 1 is active against *C. albicans* in this study. Activity against biofilm formation and preformed biofilms of *S. aureus* strains was found for compound 40 (IC_50 _= 13.51, MBC≥250, BIC_50 _= 15.43, BEC_50 _= 45.86 µg/mL), 47 (IC_50 _= 15.86, MBC≥250, BIC_50 _= 18.86, BEC_50 _= 57.62 µg/mL) and 68 (IC_50 _= 18.1, MBC≥250, BIC_50 _= 17.31, BEC_50_≥62.50 µg/mL), but not for compound 79 (IC_50_>250, MBC>250, BIC_50_>250, BEC_50_>250 µg/mL). In addition, the MS identification approach used in this study demonstrated that computer-assisted structure elucidation with UHPLC-HRMS-MS can work well for compound identifications, especially for distinguishing isomers with similar structures. Combined with chromatographic separation, it can be used for the phytochemical analysis of herbal medicine and other complex samples. The antimicrobial compounds reported here provide the chemical basis of *C. grammatus* bioactivity and support the scientific development of its quality control and therapeutic use.

## Data availability statement

The original contributions presented in the study are included in the article/**Supplementary Material**. Further inquiries can be directed to the corresponding authors.

## Ethics statement

The procedure and experimental research were approved by the Ethics and Research Committee of Gannan Medical University (approval number: 2016413). All the methods, including for experimental or field studies, and the collection of plant material, comply with relevant guidelines and regulations.

## Author contributions

Conceptualization, HbH; methodology, HbH and VT; software, HbH; validation, HbH and VT; formal analysis, HbH; investigation, HbH, BH, and HaH; data curation, HbH; writing—original draft preparation, HbH and VT; writing—review and editing, HbH, MY, AK, AKG, SKP, HaH, and WL; visualization, HbH; supervision, WL; project administration, WL and HbH; funding acquisition, WL, HbH, and HaH. All authors contributed to the article and approved the submitted version.
